# Paxillin family proteins Hic-5 and LPXN promote lipid storage by regulating the ubiquitination degradation of CIDEC

**DOI:** 10.1016/j.jbc.2023.105610

**Published:** 2023-12-28

**Authors:** Mingyu Fang, Xu Liu, Wenbo Xu, Xing Wang, Lin Xu, Tong-jin Zhao, Peng Li, Hui Yang

**Affiliations:** 1 Shanghai Key Laboratory of Metabolic Remodeling and Health, Institute of Metabolism and Integrative Biology, Fudan University, Shanghai, China; 2 Tianjian Laboratory of Advanced Biomedical Sciences, Institute of Advanced Biomedical Sciences, Zhengzhou University, Zhengzhou, Henan, China; 3 Shanghai Qi Zhi Institute, Shanghai, China

**Keywords:** Hic-5, LPXN, CIDEC, lipid droplet, ubiquitination

## Abstract

Many metabolic diseases are caused by disorders of lipid homeostasis. CIDEC, a lipid droplet (LD)-associated protein, plays a critical role in controlling LD fusion and lipid storage. However, regulators of CIDEC remain largely unknown. Here, we established a homogeneous time-resolved fluorescence (HTRF)-based high-throughput screening method and identified LPXN as a positive regulatory candidate for CIDEC. LPXN and Hic-5, the members of the Paxillin family, are focal adhesion adaptor proteins that contribute to the recruitment of specific kinases and phosphatases, cofactors, and structural proteins, participating in the transduction of extracellular signals into intracellular responses. Our data showed that Hic-5 and LPXN significantly increased the protein level of CIDEC and enhanced CIDEC stability not through triacylglycerol synthesis and FAK signaling pathways. Hic-5 and LPXN reduced the ubiquitination of CIDEC and inhibited its proteasome degradation pathway. Furthermore, Hic-5 and LPXN enlarged LDs and promoted lipid storage in adipocytes. Therefore, we identified Hic-5 and LPXN as novel regulators of CIDEC. Our current findings also suggest intervention with Hic-5 and LPXN might ameliorate ectopic fat storage by enhancing the lipid storage capacity of white adipose tissues.

With changes in dietary patterns, the incidence of metabolic disorder-associated diseases is increasing worldwide (1). A range of metabolic diseases (such as obesity (2), diabetes (3), non-alcoholic fatty liver disease (4), and atherosclerosis (5), etc.) are caused by disorders of lipid homeostasis. A lipid droplet (LD) is a critical subcellular lipid storage organelle that regulates lipid homeostasis, and its dynamic change is crucial for the regulation of lipid metabolism. Accordingly, dysregulation of LDs has been associated with many metabolic diseases (6). Lipids such as fatty acids (FAs), cholesterol, and ceramides are reesterified to neutral lipids by the lipid synthesis pathway on the endoplasmic reticulum (ER), which are then stored in the LDs (7). In addition to serving as an energy reservoir, excess fatty acids are stored in LDs to avoid lipotoxicity (8).

Lipid droplets are highly dynamic and actively involved in cellular lipid homeostasis. The lipid storage capacity of LD is controlled by its growth *via* local lipid synthesis or by LD fusion (9). CIDEC is enriched at LD-LD contact sites and facilitates the fusion and growth of LDs by generating specialized pore/channel-like structures that transfer lipids from smaller LDs to larger LDs (10). CIDEC knockout (KO) mice exhibited significant lean phenotype under both normal diet and high-fat diet (HFD) and phenotypes of lipodystrophy and insulin resistance (11, 12). CIDEC can be regulated by LD resident proteins and cytoplasmic proteins. For example, Perilipin1, an LD resident protein involved in the dynamic process of lipolysis, enhances the functional activities of CIDEC after binding to the N-terminal domain of CIDEC (13). Also, acetylation of CIDEC is synergistically modulated by histone acetyltransferase P300/CBP-associated factor (PCAF) and histone deacetylase HDAC6. When the level of fatty acids was high, the synthesis of triacylglycerol (TAG) was increased, and CIDEC was more prone to interact with PCAF, resulting in the acetylation of CIDEC at K56 and inhibiting its ubiquitination modification. In the presence of low fatty acid concentrations, CIDEC strongly interacts with HDAC6, and deacetylation of CIDEC is accompanied by increased ubiquitination (14). Given the crucial role of CIDEC in LD fusion and lipid metabolism, studying its regulatory factors can further deepen our understanding of the regulation of lipid droplets and lipid metabolism. Due to the fact that the regulatory factors of CIDEC are still largely unknown, we established a high-throughput screening method based on homogeneous time-resolved fluorescence (HTRF) to discover its regulators.

In this study, we identified LPXN and Hic-5 as positive regulatory factors of CIDEC. Hic-5 and LPXN belong to the Paxillin family, which is a kind of focal adhesion protein involved in cell proliferation, migration, and differentiation (15). Our results showed that Hic-5 and LPXN significantly enhanced CIDEC stability and promoted its accumulation by reducing its ubiquitination and inhibiting its degradation pathway. Further results confirmed that Hic-5 and LPXN significantly enlarged lipid droplets, and increased the total TAG level in 3T3-L1 adipocytes.

## Results

### Screening for regulatory proteins of CIDEC

**Figure 1:**
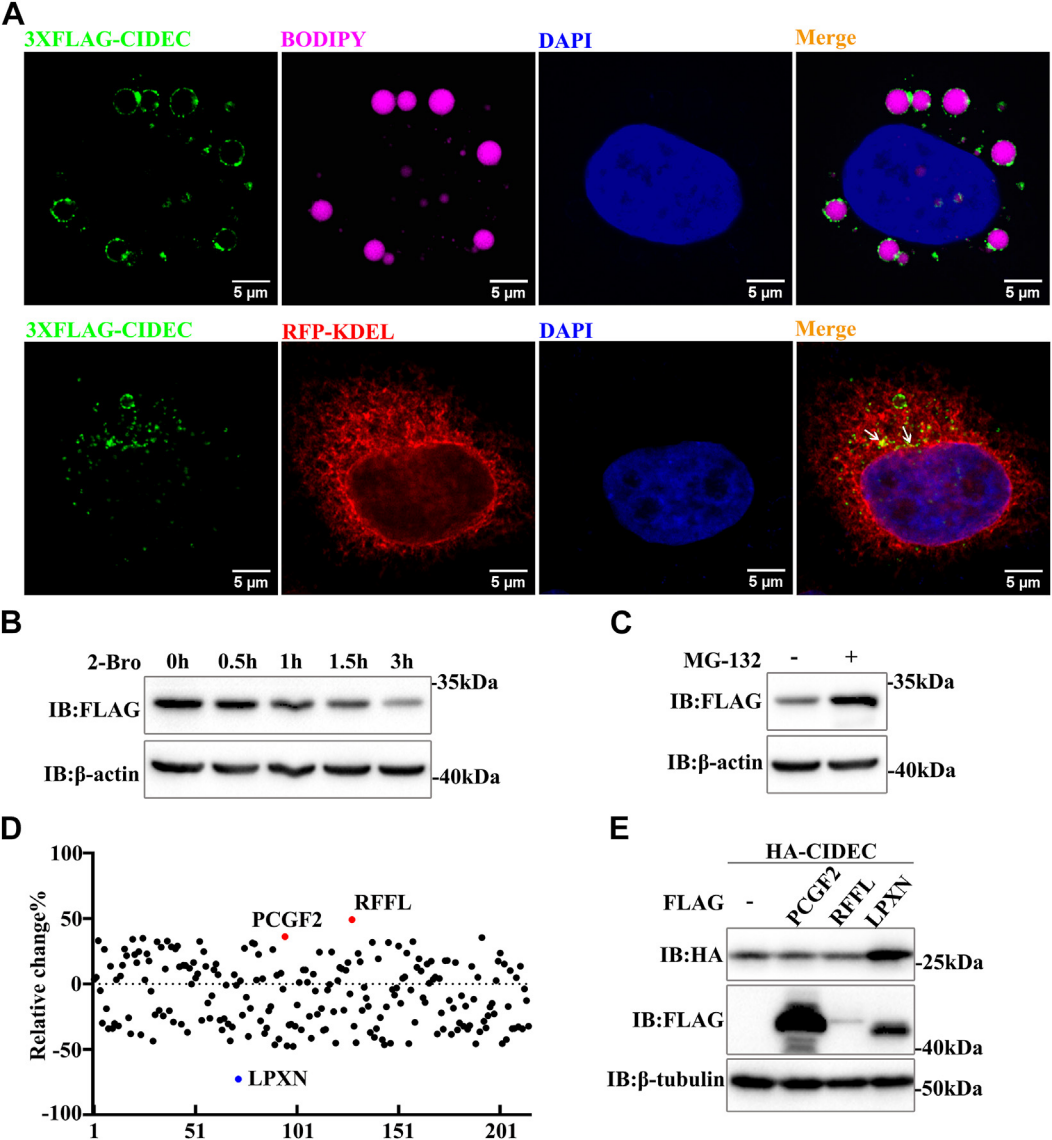
**Screening for regulatory proteins of CIDEC through the high-throughput HTRF assay**. *A*, CIDEC was localized to LD and endoplasmic reticulum (ER). 3XFLAG-CIDEC-Hela cells were pretreated with oleic acid (OA, 200 μM) for 16 h. RFP-KDEL (*red*) was used as an ER marker and transfected into 3XFLAG-CIDEC-Hela cells. 3XFLAG-CIDEC was stained with anti-FLAG antibody, LDs were stained with BODIPY 493/503 (*magenta*) and nuclei were stained with DAPI (*blue*). Scale bars, 5 μm. *B*, CIDEC accumulation was dependent on TAG synthesis. Cells were treated with 2-Bro (2.4 mM) for 0, 0.5, 1, 1.5, 3 h, and the protein level of CIDEC was detected with Western blotting. *C*, the degradation of CIDEC was dependent on the ubiquitin-proteasome pathway. Cells were harvested and evaluated with Western blotting after the addition of MG-132 (10 μM) for 2 h. *D*, the primary screening data showed three candidate proteins: PCGF2, RFFL, LPXN. PCGF2 and RFFL were negative regulatory factors (*red dot*), while LPXN was a positive regulatory protein (*blue dot*). The siRNA library that targets various proteins was transfected into 3XFLAG-CIDEC-Hela cells, and the CIDEC protein level was analyzed using an HTRF assay. *E*, HA-CIDEC was co-transfected with FLAG-PCGF2, FLAG-RFFL and FLAG-LPXN into 293T Cells. The protein levels were evaluated by Western blotting.

CIDEC promotes the fusion of lipid droplets and plays a key role in regulating lipid homeostasis, but so far few regulatory factors of CIDEC have been found. To discover novel regulatory factors of CIDEC, we have established a high-throughput screening assay based on HTRF (16). First, a 3XFLAG-CIDEC-Hela stable cell line was constructed, we examined the subcellular localization of CIDEC in this stable cell line, which was correctly localized in LDs and ER (Fig. 1*A*) (10, 17). We further verified the activity and function of CIDEC in this stable cell line. The result showed that the stability of CIDEC depends on the synthesis of TAG, and the protein level of CIDEC was significantly reduced when cells were treated with 2-bromooctanoate (2-Bro), an inhibitor that can inhibit the activity of DGAT and block the synthesis of TAG (Fig. 1*B*) (18, 19). Meanwhile, when cells were treated with MG-132 (a proteasome inhibitor), CIDEC accumulated significantly, indicating that CIDEC was dependent on the ubiquitination-proteasome degradation pathway (Fig. 1*C*). Next, we performed a high-throughput screening process based on the combination of the small interfering RNA (siRNA) library (targeting 215 genes) and HTRF. We identified three candidate proteins that might be novel regulators of CIDEC: PCGF2, RFFL, and LPXN. Among them, PCGF2 and RFFL might be negative regulators of CIDEC, while LPXN was a positive regulator of CIDEC (Fig. 1*D* and Table S1). To further validate the results of high-throughput screening, CIDEC and these candidate proteins were co-expressed in 293T cells, and Western blotting data showed that LPXN significantly increased the protein level of CIDEC (Fig. 1*E*). However, PCGF and RFFL did not affect the protein level of CIDEC (Figs. 1*E* and S1), which we speculated might be due to the insufficiently significant regulatory effects of PCGF and RFFL on CIDEC in the high-throughput screening result (less than 50%) or possible off-target effects.

### LPXN is a novel regulator of CIDEC by enhancing its stability

**Figure 2:**
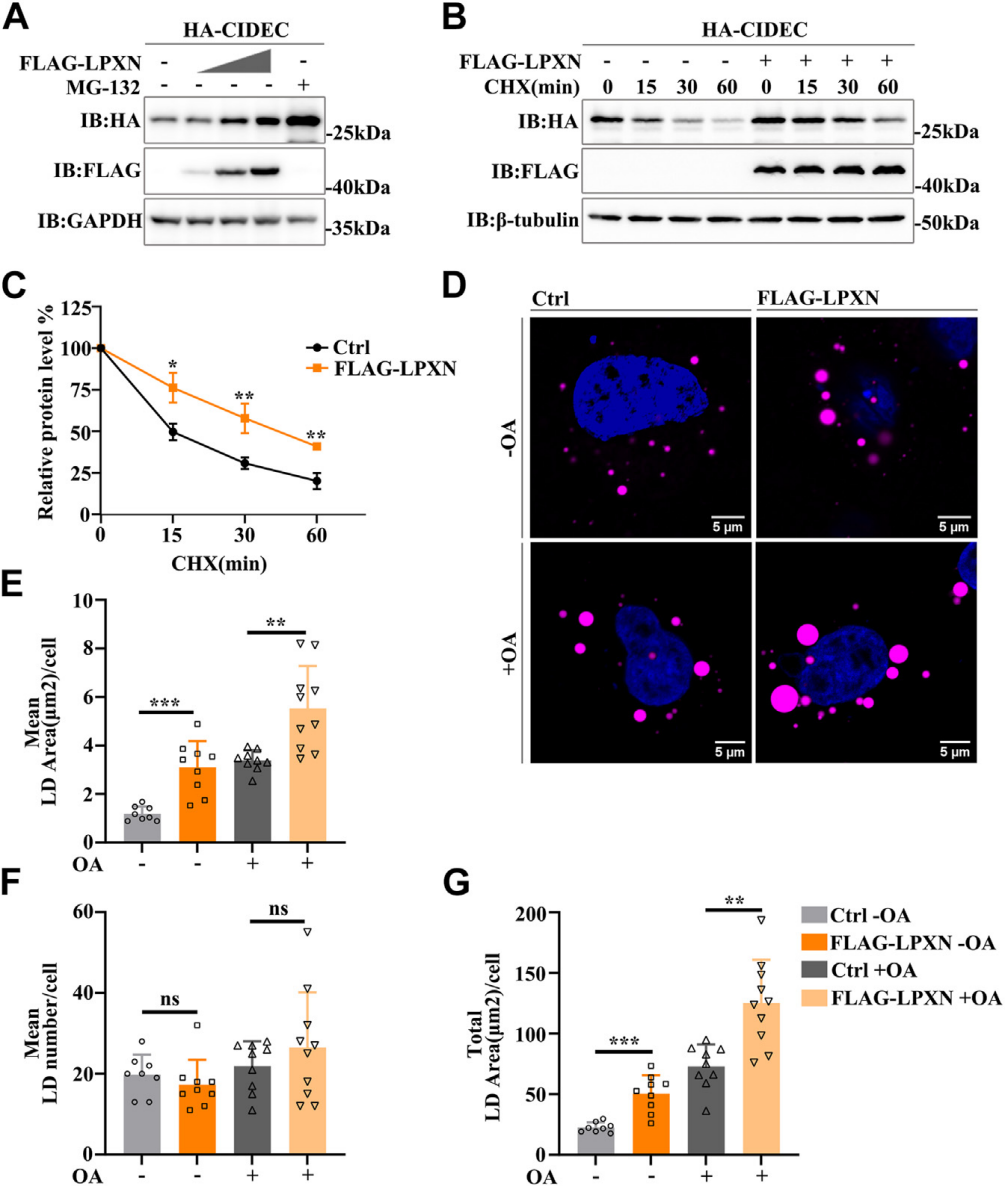
**LPXN enhances CIDEC stability**. *A*, effect of LPXN on the protein level of CIDEC. HA-CIDEC was co-transfected with different amounts of FLAG-LPXN into 293T Cells. Cells were harvested and the protein level was evaluated by Western blotting. Cells were treated with MG-132(10 μM) for 2 h as a positive control. IB, immunoblotting. *B*, effect of LPXN on the stability of CIDEC. HA-CIDEC was co-transfected with or without FLAG-LPXN into 293T cells. Cells were harvested 0, 15, 30, or 60 min after the addition of cycloheximide (CHX, 100 μg/ml). *C*, Quantitative analysis of the relative level of CIDEC based on the results in (*B*). Experiments were repeated three times(A Student’s two-tailed *t* test (unpaired)) for statistical analysis (∗*p* ＜ 0.05; ∗∗∗*p* ＜ 0.001; n = 3). *D*, Effect of LPXN on LDs. Representative LD images in 3XFLAG-CIDEC-Hela cells transfected with or without FLAG-LPXN were captured, respectively. OA (200 μM) was added to the culture medium for 16 h. LDs were stained with BODIPY 665 (*magenta*), nuclei were stained with DAPI (*blue*). Scale bars, 5 μm. *E–G*, Quantitative analysis of LDs size (*E*), LDs number (*F*), and total LDs size in cells. *G*, images were collected from 10 cells in each group for statistical analysis (A Student’s two-tailed *t* test (unpaired); ns, no significant difference; ∗*p* ＜ 0.05; ∗∗∗*p* ＜ 0.001).

Then, we conducted a dose-dependent experiment to confirm the impact of LPXN on CIDEC and found that the protein level of CIDEC was accumulated with the increase of LPXN (Fig. 2*A*). We further tested the effect of LPXN on the stability of CIDEC. It has been reported that the half-life of CIDEC protein is relatively short (17). Our results showed that the half-life of CIDEC was increased to about 45 min after overexpression of LPXN, indicating that LPXN could increase CIDEC stability (Fig. 2, *B* and *C*). As CIDEC can promote LD fusion, and enlarge the volume of lipid droplets, thereby increasing the lipid storage capacity of cells (10), we tested whether LPXN affected the lipid droplet phenotype. When LPXN was overexpressed in 3XFLAG-CIDEC-Hela, the mean area of individual lipid droplets in the cells increased significantly, while the number of lipid droplets decreased very slightly, and the total area of LDs increased significantly (Fig. 2, *D*–*G*). Meanwhile, when oleic acid (OA) was added, the same LD phenotype was observed after overexpression of LPXN (Fig. 2, *D*–*G*). In conclusion, LPXN was a novel regulator of CIDEC, which promoted the accumulation and stability of CIDEC and altered the size of LDs.

### Homologous protein Hic-5 also enhances CIDEC stability

**Figure 3:**
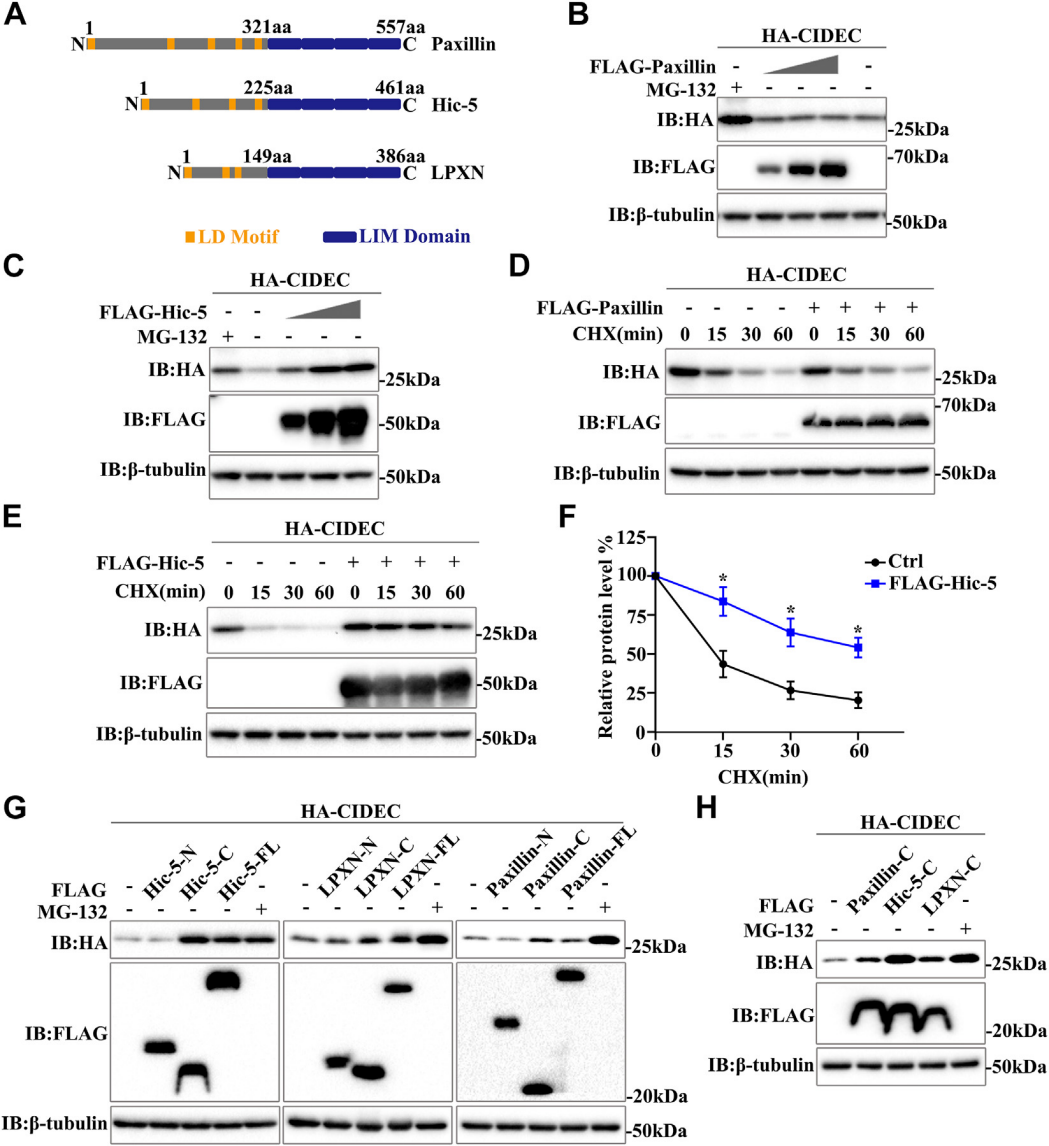
**Hic-5 also enhances CIDEC stability**. *A*, domain architecture of the Paxillin family members. Paxillin is a 557-amino acid protein comprised of multiple structural domains including five leucine-rich LD motifs and four LIM domains. Hic-5 contains four LD motifs and LPXN includes three LD motifs. All three members exhibit extensive homology within the C-terminal LIM domains. *B* and *C*, effect of Paxillin or Hic-5 on the protein level of CIDEC. HA-CIDEC was co-transfected with different amounts of FLAG-Paxillin or FLAG-Hic-5 into 293T Cells. The protein level was analyzed by Western blotting. *D*, effect of Paxillin on CIDEC stability. HA-CIDEC was co-transfected with or without FLAG-Paxillin into 293T cells. Cells were treated with cycloheximide (CHX, 100 μg/ml) for 0, 15, 30, and 60 min. Western blotting was used to detect the protein level. *E*, Effect of Hic-5 on the stability of CIDEC. HA-CIDEC was co-transfected with or without FLAG-Hic-5 into 293T cells. Cells were harvested 0, 15, 30, or 60 min after the addition of CHX (100 μg/ml). *F*, quantitative analysis of the relative level of CIDEC from (*E*) (A Student’s two-tailed *t* test (unpaired)) for statistical analysis (∗*p* ＜ 0.05; ∗∗∗*p* ＜ 0.001; n = 3). *G*, effect of the truncations of Paxillin family members on the protein level of CIDEC. HA-CIDEC was co-transfected with the N-terminus, C-terminus, or Full length of FLAG-Paxillin or FLAG-Hic-5 or FLAG-LPXN into 293T Cells. The protein level was analyzed by Western blotting. *H*, effect of the C-terminus of Paxillin family members on the protein level of CIDEC. HA-CIDEC was co-transfected with the C-terminus of FLAG-Paxillin or FLAG-Hic-5 or FLAG-LPXN into 293T Cells. The protein level was analyzed by Western blotting.

LPXN belongs to the Paxillin family, which has two other members: Paxillin and Hic-5. Many previous studies have shown that Paxillin family is a kind of focal adhesion protein (20). As a scaffold protein, Paxillin family interacts with many different focal adhesion-related proteins and participates in a variety of biological processes such as cell adhesion, migration, differentiation and signal transduction (21). Paxillin is widely expressed in various tissues (22), and Hic-5 is mainly expressed in smooth muscle cells, fibroblasts, and platelets (23). LPXN is highly expressed in immune cells such as T cells, B cells, and macrophages (24). In addition, some studies have shown that LPXN is also expressed in prostate cells (25), bladder cancer cells (26), and smooth muscle cells (27). The discovery of the Paxillin family is due to the similarity in protein structures among the three members, all of which contain several short leucine and aspartic acid-rich (LD) motifs on the N-terminal and are involved in multiple protein-protein interactions as a molecular adapter/scaffold protein (28). The C-terminal contains four highly conserved LIM domains, which are double zinc finger motifs. It is involved in the localization of focal adhesion and regulates the assembly of microfilaments together with other focal adhesion proteins (Fig. 3*A*) (29). At present, the research of Paxillin family proteins mainly focuses on the direction related to cancer cell migration, with little research on metabolism. Our previous data showed that LPXN could increase the stability of CIDEC, indicating that LPXN was involved in lipid metabolism. Then we examined whether the other two Paxillin family members also affected CIDEC stability. The result showed that overexpression of Paxillin did not lead to the accumulation of CIDEC (Fig. 3*B*). However, CIDEC gradually accumulated with the increase of Hic-5 (Fig. 3*C*). Meanwhile, the results of the CHX chase assay showed that the half-life of CIDEC protein did not change when Paxillin was overexpressed (Fig. 3*D*). Importantly, Hic-5 also had a significant effect on the stability of CIDEC (Fig. 3, *E* and *F*). In short, these data suggested that Paxillin did not affect the stability of CIDEC, whereas both Hic-5 and LPXN could enhance the stability of CIDEC. In addition, we further investigated which domains of Hic-5 and LPXN were required for promoting CIDEC accumulation. The results showed that the C-terminus of Hic-5 and LPXN promoted CIDEC accumulation, while the N-terminus of them did not lead to accumulation (Fig. 3, *G* and *H*). Meanwhile, when the C-terminus of Paxillin was overexpressed, the protein level of CIDEC increased (Fig. 3, *G* and *H*). However, overexpression of full-length and N-terminal Paxillin did not alter the protein level of CIDEC, so we suspected that the N-terminus of Paxillin might have an inhibitory effect on its C-terminus promoting CIDEC accumulation (Fig. 3, *G* and *H*).

### Effects of Hic-5 and LPXN on CIDEC stability are independent of TAG synthesis and FAK signaling pathway

**Figure 4:**
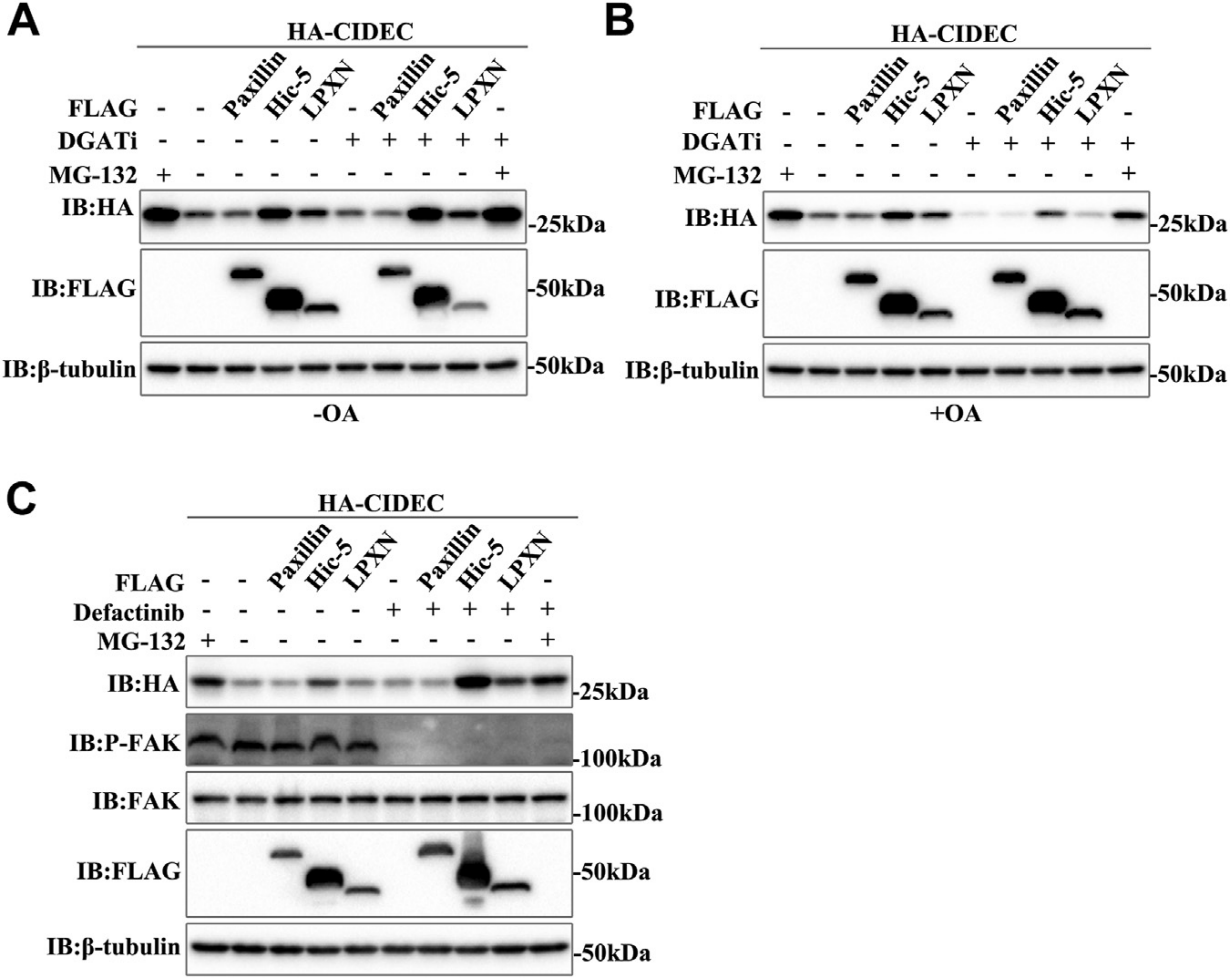
**Effects of Hic-5 and LPXN on CIDEC stability are independent of TAG synthesis and FAK signaling pathway**. *A* and *B*, Hic-5 and LPXN increased the stability of CIDEC independent of TAG synthesis. HA-CIDEC was co-transfected with FLAG-Paxillin, FLAG-Hic-5 or FLAG-LPXN into 293T cells. Cells were treated with DGAT inhibitor and OA (200 μM) for 18 h and harvested to determine the CIDEC protein level. *C,* Hic-5 and LPXN did not enhance CIDEC stability through the FAK signaling pathway. HA-CIDEC was co-transfected with FLAG-Paxillin, FLAG-Hic-5, or FLAG-LPXN into 293T cells and Defactinib (2 μM) was added to culture medium for 24 h. The protein level was analyzed by Western blotting.

Next, we further investigated how Hic-5 and LPXN enhanced the stability of CIDEC. Since the accumulation of CIDEC was dependent on the synthesis of TAG (17), we first examined whether they promote the stability of CIDEC by increasing TAG synthesis. In the absence of OA treatment, the synthesis of TAG was blocked and the protein level of CIDEC was slightly reduced in the presence of DGAT inhibitor. Overexpression of Hic-5 and LPXN still resulted in a significant accumulation of CIDEC, indicating that blocking TAG synthesis did not alter their regulatory effects on CIDEC (Fig. 4*A*). When OA was added, the TAG synthesis process became more active. After blocking TAG synthesis, although CIDEC was significantly reduced, Hic-5 and LPXN also increased the protein levels of CIDEC compared with the control group (Fig. 4*B*).

In addition, as a scaffold protein, the Paxillin family can interact with a variety of proteins. Focal adhesion kinase (FAK), as one of the most important interacting proteins of Paxillin family, is a cytoplasmic non-receptor protein tyrosine kinase (30). By integrating a variety of extracellular signaling molecules, it is activated by self-phosphorylation, and activated FAK binds to a variety of downstream factors to phosphorylate and activate downstream signaling pathways, resulting in changes in metabolism, proliferation, migration, and other processes (31, 32, 33). Next, we investigated whether Hic-5 and LPXN induced downstream signal changes through the FAK signaling pathway, leading to the accumulation of CIDEC. Defactinib, a specific inhibitor of FAK, inhibits the phosphorylation of FAK Tyr397 (34). When Defactinib was added, the phosphorylation level of FAK (Tyr397) was significantly reduced, but the protein level of CIDEC was not reduced. More interestingly, compared with no defactinib treatment, Hic-5 and LPXN promoted the accumulation of CIDEC more significantly (Fig. 4*C*). In conclusion, Hic-5 and LPXN did not promote CIDEC stability through TAG synthesis and FAK signaling pathways.

### Hic-5 and LPXN promote CIDEC stability through the ubiquitin-proteasome pathway

**Figure 5:**
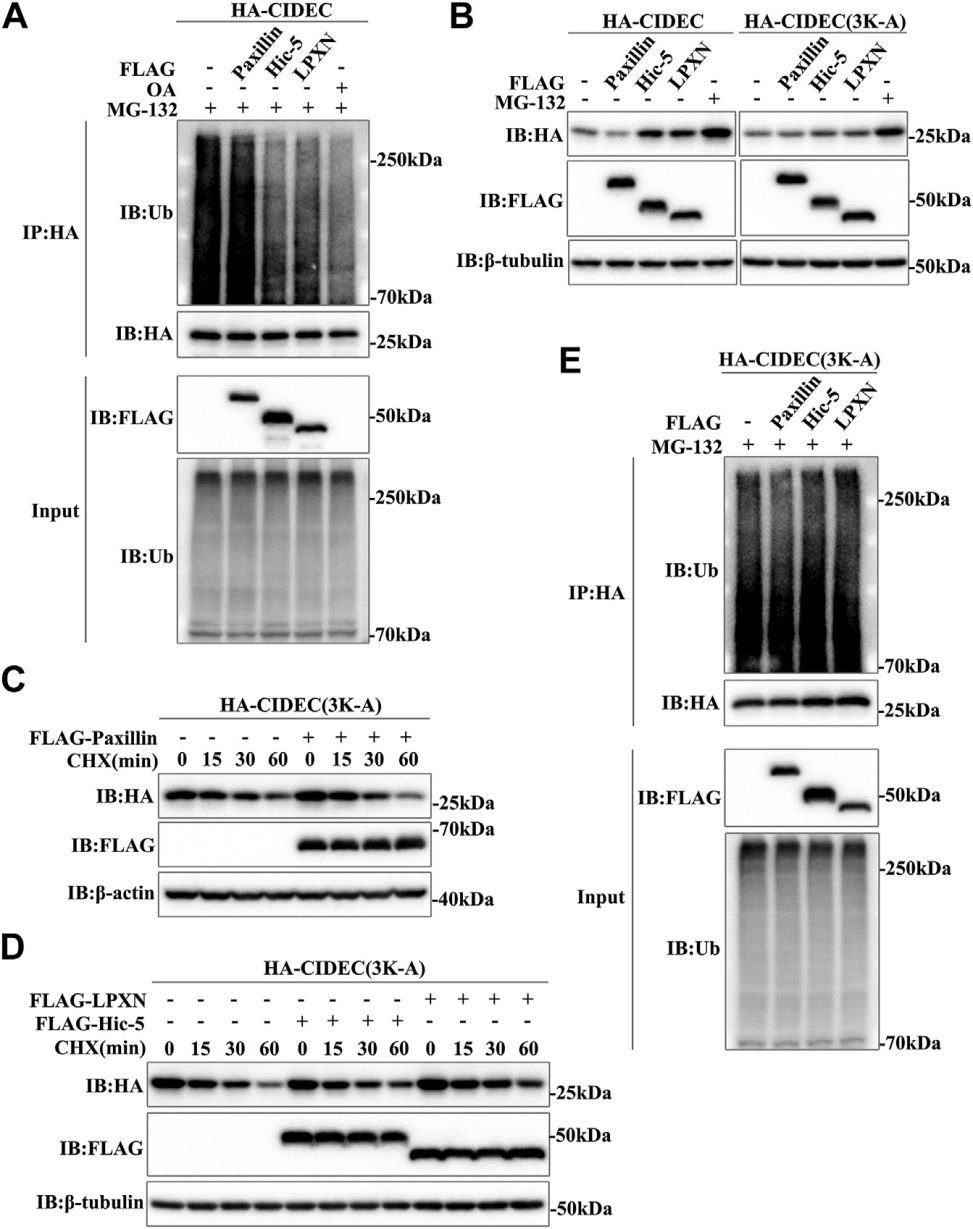
**Hic-5 and LPXN promote the accumulation of CIDEC by regulating its ubiquitination.***A*, effect of Paxillin family Hic-5 and LPXN on ubiquitination of CIDEC. HA-CIDEC was co-transfected with FLAG-Paxillin, FLAG-Hic-5 or FLAG-LPXN into 293T cells. MG132 was added for 2 h to inhibit ubiquitination-proteasome degradation. Cells treated with or without OA (200 μM) for 16 h were immunoprecipitated with anti-HA agarose beads. Anti-Ubiquitin (Ub) antibody was used to detect the ubiquitination level of CIDEC. IP, immunoprecipitation. *B*, effect of Paxillin family on the protein level of CIDEC(3K-A). HA- CIDEC or CIDEC(3K-A) was co-transfected with FLAG-Paxillin, FLAG-Hic-5, or FLAG-LPXN into 293T Cells. The protein level was analyzed by Western blotting. *C* and *D*, effect of Paxillin family on the stability of CIDEC(3K-A). HA-CIDEC(3K-A) was co-transfected with FLAG-Paxillin, FLAG-Hic-5 or FLAG-LPXN into 293T Cells. Cells were harvested 0, 15, 30, or 60 min after the addition of CHX (100 μg/ml). *E*, effect of Paxillin family on ubiquitination level of CIDEC(3K-A). HA-CIDEC(3K-A) was co-transfected with FLAG-Paxillin, FLAG-Hic-5 or FLAG-LPXN into 293T cells. Cells were immunoprecipitated with anti-HA agarose beads after MG-132 treatment for 2 h. anti-Ub antibody was used to detect the ubiquitination level of CIDEC(3K-A).

The degradation of CIDEC is dependent on the ubiquitination-proteasome pathway (17). Next, we detected whether Hic-5 and LPXN led to the accumulation of CIDEC by inhibiting the ubiquitination of CIDEC. The ubiquitination level of CIDEC was significantly reduced when treated with OA, as a positive control. When Paxillin was overexpressed, the ubiquitination level of CIDEC did not change, while Hic-5 and LPXN resulted in a significant decrease of the ubiquitinated CIDEC (Fig. 5*A*). The C-terminal of CIDEC contains three highly conserved lysines (Lys-223, Lys-225, and Lys-235), which are key ubiquitination modification sites (17). When all three lysines of CIDEC were mutated to alanine (3K-A), Hic-5 and LPXN did not result in a significant accumulation of CIDEC (3K-A) (Fig. 5*B*). Meanwhile, we examined the effect of Paxillin family members on the half-life of CIDEC (3K-A). Compared with wild-type CIDEC, CIDEC (3K-A) has a half-life of nearly 60 min, and its stability is significantly enhanced. Our data showed that Hic-5 or LPXN did not enhance the stability of CIDEC(3K-A) (Fig. 5, *C* and *D*). In addition, the ubiquitination of CIDEC (3K-A) was not regulated by Hic-5 or LPXN (Fig. 5*E*). In summary, these results suggested that Hic-5 and LPXN enhanced the stability of CIDEC by reducing its ubiquitination and inhibiting its proteasome degradation pathway.

### Hic-5 and LPXN promote lipid storage in adipocytes

**Figure 6:**
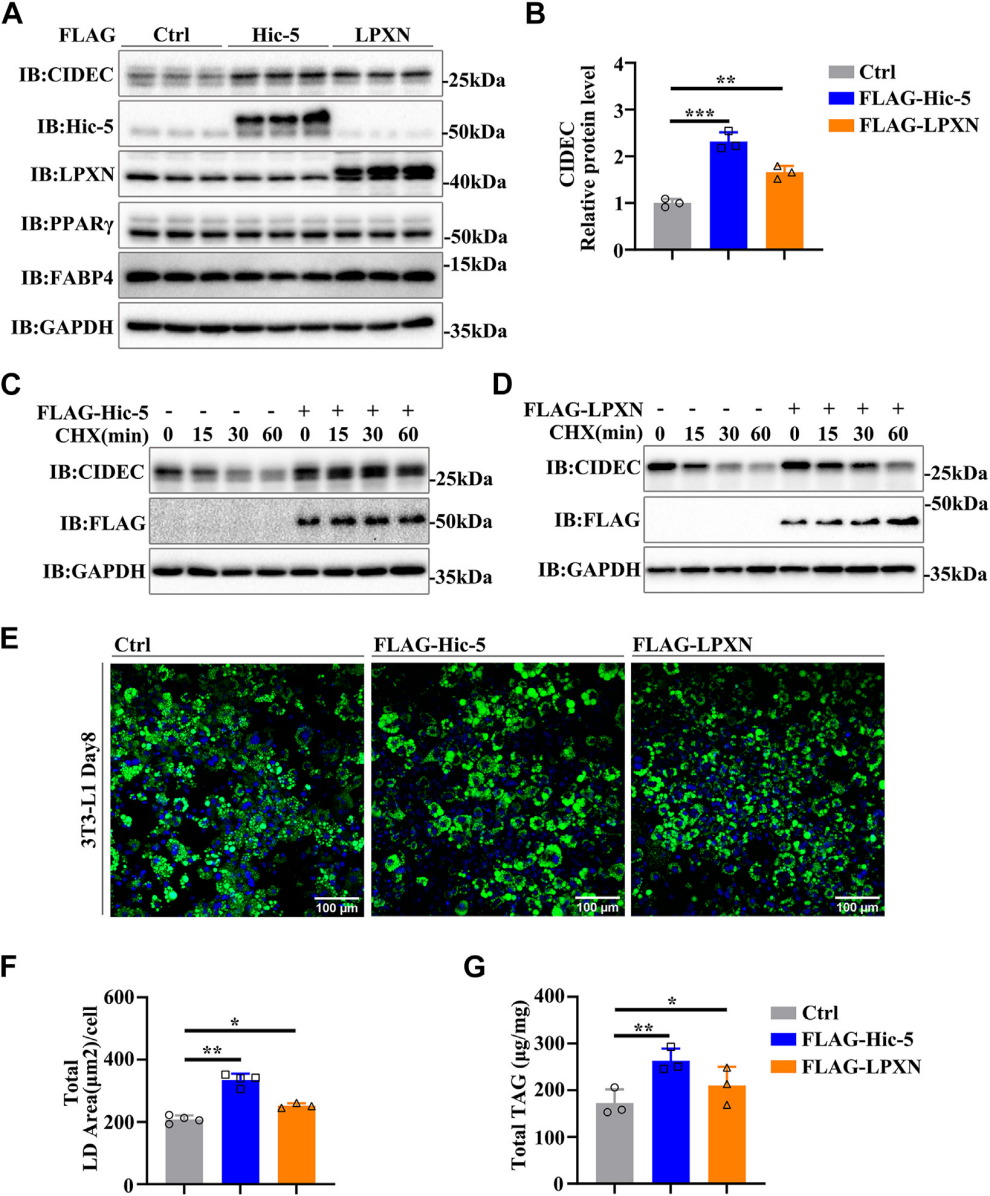
**Effect of Paxillin family on the CIDEC stability and lipid storage in adipocytes.***A*, effect of Hic-5 and LPXN on the protein level of CIDEC. 3T3-L1 cells were infected with lentivirus encoding FLAG-tagged Hic-5 or LPXN and then differentiated for 8 days. IB, immunoblotting. *B*, quantitative analysis of the relative level of CIDEC based on the results in (*A*). (A Student’s two-tailed *t* test (unpaired)) for statistical analysis (∗*p* ＜ 0.05; ∗∗∗*p* ＜ 0.001; n = 3). *C* and *D*, effect of Hic-5 and LPXN on the CIDEC stability. Differentiated 3T3-L1 cells (day 6 after induction) were infected with lentivirus encoding FLAG-tagged Hic-5 or LPXN for 2 days. Cells were harvested and the protein level was evaluated with Western blotting after the addition of CHX (100 μg/ml) for 0, 15, 30, or 60 min. *E*, Hic-5 and LPXN increase total LD size in adipocytes. LDs were stained with BODIPY 493/503 (*green*) and nuclei were stained with DAPI (*blue*). Scale bars, 100 μm. *F*, quantitative analysis of LDs size in cells (*E*). Images were collected in each group for statistical analysis (A Student’s two-tailed *t* test (unpaired); ns, no significant difference; ∗*p* ＜ 0.05; ∗∗∗*p* ＜ 0.001. n = 4). *G*, effect of Hic-5 and LPXN on the total TAG level in adipocytes. Differentiated adipocytes transfected with FLAG-tagged Hic-5 or LPXN were harvested to extract total TAG (A Student’s two-tailed *t* test (unpaired); ns, no significant difference; ∗*p* ＜ 0.05; ∗∗∗*p* ＜ 0.001. n = 3).

As a key LD regulatory protein, CIDEC is highly expressed in white adipocytes and regulates the LD fusion process (35). To determine whether Hic-5 and LPXN can alter the LD phenotype by promoting the stability of CIDEC in adipocytes, Paxillin family members were overexpressed into 3T3-L1 adipocytes. The data showed that both Hic-5 and LPXN resulted in a significant up-regulation of CIDEC with PPARγ and FABP4 as adipocyte differentiation markers (Figs. 6, *A* and *B* and S2, *A* and *B*). Furthermore, we examined the effect of Paxillin family members on the stability of CIDEC in adipocytes, and the result showed that Paxillin did not alter the half-life of CIDEC (Fig. S2, *C* and *D*). However, compared with the control group, Hic-5 and LPXN significantly increased the half-life of CIDEC (Figs. 6, *C* and *D* and S2, *E* and *F*), confirming that both Hic-5 and LPXN can promote CIDEC accumulation in adipocytes. Subsequently, the LD phenotype in differentiated 3T3-L1 adipocytes was also detected. As expected, both Hic-5 and LPXN significantly increased the LD area in adipocytes (Fig. 6, *E* and *F*). Lipid droplets are important subcellular organelles for TAG storage. We detected total TAG levels in adipocytes, and the results showed that Hic-5 and LPXN significantly increased the content of TAG in adipocytes (Fig. 6*G*). In short, Hic-5 and LPXN promoted lipid storage by directly targeting the stability of CIDEC.

Moreover, we examined the subcellular localization of Hic-5 and LPXN in adipocytes. The data showed that Hic-5 and LPXN were localized in cytosol, nucleus and focal adhesion, and LPXN was more distributed in the nucleus (Fig. S3, *A* and *B*). Previous research showed that the protein levels of CIDEC were significantly increased in white adipose tissue of obese mice or humans (14). So we analyzed expression changes of Hic-5 and LPXN in the diet-induced obesity model. Transcriptomics profiling (GEO: GSE182930) of white adipose tissues of wild-type mice fed a normal chow diet (NCD) or high-fat diet (HFD) were analyzed in the study (36). The data showed that both the expression levels of Hic-5 and LPXN were upregulated in white adipose tissues of diet-induced obesity mice (Fig. S3, *C* and *D*), implying that Hic-5 and LPXN may promote the stability of CIDEC by regulating its ubiquitination or deubiquitination during the development of obesity.

## Discussion

CIDE family proteins regulate LDs fusion and lipid storage, and play an important role in metabolic diseases such as obesity and fatty liver. Here, we focused on identifying regulatory factors of CIDEC and found that Paxillin family protein Hic5 and LPXN promoted lipid storage by regulating the ubiquitination degradation of CIDEC.

First, we set up a high-throughput screening system *in vitro*. After constructing a stable 3XFLAG-CIDEC HeLa cell line, we combined HTRF, a very simple and sensitive method to screen the regulatory factors of CIDEC. We identified three potential regulators: PCGF2, RFFL, and LPXN by this efficient high-throughput screening system. Further validation results showed that LPXN could indeed positively regulate CIDEC, indicating that this screening system was effective and could be applied to a wider range of screening. Next, we demonstrated that LPXN could enhance the stability and accumulation of CIDEC, leading to larger lipid droplets. Given that LPXN is a member of the Paxillin family, we investigated whether two other homologous proteins, Paxillin and Hic-5, may also regulate CIDEC. The results showed that Paxillin did not change the protein level of CIDEC, but Hic-5 could induce significant accumulation of CIDEC and enhance its stability. This is the first time that Paxillin family protein Hic-5 and LPXN were linked to LD regulation, implying that they also participated in lipid metabolism.

Next, to figure out how Hic-5 and LPXN promoted CIDEC accumulation, we first blocked TAG synthesis with a DGAT inhibitor, as CIDEC stability is regulated by TAG synthesis. However, Hic-5 and LPXN can still enhance CIDEC stability in the presence of DGAT inhibitor, which indicated that their effect of them on CIDEC was not regulated through TAG synthesis. Considering that the Paxillin family interacts with FAK, a very important focal adhesion protein kinase, we hypothesized that Hic-5 and LPXN could promote CIDEC accumulation through FAK and its downstream signaling pathways. Defactinib was used to block FAK phosphorylation, thereby inhibiting the activation of FAK downstream signaling pathways. Unexpectedly, when FAK was inhibited, CIDEC was still regulated by Hic-5 and LPXN, and the effect was more significant, which might be due to the increase of Hic-5 and LPXN or the inhibition of FAK and its downstream signaling pathways (PI3K/AKT, JNK, ERK1/2) indirectly affects the activities or protein levels of factors regulating the ubiquitination of CIDEC mediated by Hic-5 or LPXN. As a protein with a short half-life, CIDEC relies on the ubiquitination-proteasome degradation pathway. We found that Hic-5 and LPXN can significantly reduce the ubiquitination level of CIDEC. When the three key conserved ubiquitination sites in the C terminus of CIDEC were mutated, Hic-5 and LPXN could not promote its accumulation, and CHX chase experiments also showed that they also failed to enhance the stability of CIDEC (3K-A). Meanwhile, the ubiquitination of CIDEC (3K-A) mutants was not regulated by Hic-5 and LPXN. These results confirmed that Hic-5 and LPXN enhanced the stability of CIDEC by inhibiting its ubiquitination degradation pathway, but how they regulated the ubiquitination of CIDEC needs to be further elucidated.

Finally, we overexpressed Paxillin family proteins in adipocytes with high expression of CIDEC to detect the changes in LDs and the ability of lipid storage in adipocytes. Notably, both Hic-5 and LPXN promoted CIDEC accumulation in 3T3-L1 adipocytes and increased the volume of lipid droplets and total TAG in adipocytes. Overall, we characterized the regulatory role of Paxillin family on CIDEC and revealed that Hic-5 and LPXN can promote CIDEC stability by inhibiting its ubiquitination. The development process of obesity, non-alcoholic fatty liver disease, and other lipid metabolism–related metabolic syndrome, is accompanied by a certain degree of fat ectopic storage (37). Excessive lipids will produce lipotoxicity, forming a vicious cycle, and this is closely related to the fat storage capacity of adipose tissue (38). Hic-5 and LPXN can promote the accumulation of CIDEC, enable adipocytes to store more lipids, and may avoid more fat ectopic storage, thereby alleviating fatty liver and other metabolic diseases caused by obesity.

## Experimental procedures

### Plasmid construction

Full-length cDNA encoding various proteins (CIDEC, PCGF2, RFFL, LPXN, Paxillin, Hic-5, and related truncations) was inserted into pcDNA3.1-FLAG, pcDNA3.1-HA, or lentivirus vector (CD513B). Amino acid substitutions on CIDEC were generated from wild-type CIDEC by PCR site-directed mutagenesis (PrimeSTAR Max DNA Polymerase, Takara, R045A). The integrity of all plasmid DNA constructs was verified by sequencing analysis (Tsingke Biotechnology Co, Ltd).

### Cell culture and transfection

HEK293T cells, HeLa cells, and 3T3-L1 preadipocytes were maintained in Dulbecco’s modified Eagle’s medium (MACGENE, CM10013) containing 10% bovine serum (VivaCell, C04001). The differentiation of the 3T3-L1 preadipocytes was initiated 2 days post-confluence by the addition of 5 mg/ml insulin (Sigma, I5500), 1 M dexamethasone (Sigma, D4902), and 0.5 mM isobutylmethylxanthine (Sigma, I5879). The differentiation medium was subsequently replaced with Dulbecco’s modified Eagle’s medium/fetal bovine serum (Gibco, 10091-148) supplemented with only 5 mg/ml insulin for 4 days. Cells were used for experiments 8 days after differentiation. HEK293T cells or HeLa cells were transfected using polyJet (SignaGen Laboratories, SL100688) according to the manufacturer’s instruction.

### Lentivirus preparation and construction of stable cell lines

CIDEC-3XFLAG was inserted into CD513B expression vector and packaged into a lentivirus, as previously described (39). The culture medium containing virus particles was filtered through a 0.45 μm filter (Millipore, SLHPR33RB) and frozen at 80 °C until further use. HeLa cells were infected for 24 h with the lentivirus expressing CIDEC-3XFLAG, and then cultured in a medium containing an appropriate concentration of Puromycin (InvivoGen, ant-pr-1) for 3 to 4 days. Positive cells were seeded at very low concentrations to allow the picking of single-cell clones. Clones were checked for the presence of CIDEC by Western blotting. 3T3-L1 preadipocytes were infected for 24 h with the lentivirus expressing FLAG-Paxillin, FLAG-Hic-5, or FLAG-LPXN. Differentiation was induced in infected preadipocytes. A lentivirus generated from the empty vector was used as a negative control.

### Immunofluorescent staining and LD evaluation

In brief, cells were rinsed twice in PBS and fixed with 4% PFA for 20 min. Subsequently, the cells were permeabilized with 0.3% Triton-X100 in PBS containing 1% BSA for 1h. A primary antibody against Flag (CST, D6W5B), Hic-5 (BD Biosciences, 611164), or LPXN (ABclonal, A9803) was added at a dilution of 1:400 at 4 °C overnight. Cells were washed three times with PBS, and incubated with fluorescently labeled secondary antibody (Invitrogen, A11008) at a dilution of 1:400 for 1 h followed by DAPI (Invitrogen, D3571), BODIPY, and phalloidin (Yeasen, 40734ES75), as indicated. Images for morphological analysis were acquired with ZEISS LSM 880 laser confocal microscope using a 63× oil or 25× air immersion objective. For quantitative analysis of cells containing LDs, 3XFLAG-CIDEC-Hela transfected with indicated plasmids were fixed and stained with BODIPY 665 (Invitrogen, D3932), differentiated 3T3-L1 adipocytes infected with indicated lentiviruses were stained with BODIPY 493/503 (Invitrogen, D3922). The area and number of LDs were measured for each group using IMARIS Viewer software.

### HTRF assay

3XFLAG-CIDEC HeLa cells were transfected with siRNA library oligos using Lipofectamine RNAiMAX (Invitrogen, 13778075) according to the manufacturer’s instructions in 384-well plates. Cells were lysed after the addition of HTRF buffer (50 mM NaH_2_PO_4_, 0.1% BSA, 400 mM NaF, 0.05% Tween-20, 2% TritonX-100), and FLAG Check kit (cisbio, 63ADK036PEG) was used to detect 3XFLAG-CIDEC protein level according to the manufacturer’s instructions. The Quant-iT PicoGreen dsDNA quantitative kit (Invitrogen, P11495) was performed to standardize the cell number per well. A microplate reader (Tecan Spark) was used for all measurements.

### Immunoprecipitation and Western blotting

HEK293T cells were lysed for 30 min in RIPA buffer (50 mM Tris-HCl, pH 8.0, 150 mM NaCl, 1% TritonX-100, 1 mM EDTA and protease inhibitors (Roche, EDTA-free, 04693159001)). Supernatant after spinning (15 min at 13,000*g*) was incubated with anti-HA or anti-FLAG agarose beads (Smart-Lifesciences, SA068005/SA042010) for 4 h at 4 °C. Beads were washed four times before the addition of SDS loading buffer containing 250 mM Tris-HCl (pH 6.8), 10% SDS, 50% glycerol, 0.075% bromophenol blue, 500 mM DTT followed by 10 min incubation at 95 °C. For ubiquitination detection, beads were washed with wash buffer containing 500 mM NaCl additionally. Subsequently, immunoprecipitated proteins were separated by SDS-PAGE for Western blotting. In brief, samples were separated by a 10% acrylamide gel and transferred to PVDF membrane (Immobilon-P, 0.45 μm, Millipore, IPVH00010) at 300 mA for 2 h. The filters were blocked in TBST containing 5% defatted milk and incubated with a primary antibody at 4 °C overnight, washed 4 times for 6 min, and incubated with the secondary antibody for 60 min. The filter was incubated with ECL reagent (Thermo Scientific SuperSignal West Pico PLUS, Thermo Scientific, 34580) and the signal was detected using the Chemidoc XRS+ imager (Bio-Rad, ChemiDoc XRS). The intensity of bands was quantified using ImageJ software. Antibodies against HA (C29F4), FLAG (D65WB), and PPARγ (81B8) were purchased from Cell Signaling Technology (CST). The antibody against β-actin (A5441) was purchased from Sigma. Antibodies against GAPDH (AC001), β-tubulin (AC008), FABP4 (A11481), and LPXN (A9803) were purchased from ABclonal. The antibody against Paxillin (ab32084) was purchased from Abcam. The antibody against Hic-5 (611164) was purchased from BD Biosciences. A rabbit polyclonal antibody against mouse CIDEC was raised against Fsp27 aa 1 to 190 (40).

### Cellular TAG measurement

For determination of cellular TAG levels, lipids were extracted from 8-days differentiated 3T3-L1 adipocytes as previously described (41). Briefly, methanol (1.5 ml) was added to a 200 μl sample aliquot, which was placed into a glass tube. Then, 4.5 ml of MTBE (Sigma, 35875) was added and the mixture was vortexed for 1 min and incubated for 1 h at room temperature in a shaker. After adding 1.25 ml of water. the sample was centrifuged at 1000*g* for 10 min. The upper (organic) phase was collected and dried in a vacuum centrifuge. The lipids were dissolved in 5% NP-40 and determined by Triglyceride LabAssay (Wako, 290-63701). The lower phase was centrifuged at 4000*g* for 20 min, and the pellets were resuspended in 0.2 M KOH buffer for protein quantification. TAG levels were normalized to the protein concentration of each sample.

### Statistical analysis

Data were subjected to statistical analysis and plotted using GraphPad Prism 8.0. To statistically analyze the significant difference between two parameters, a two-tailed Student’s *t* test (unpaired) was used. Results were reported by mean ± SD

## Data availability

Data will be made available on request.

## Supporting information

The article contains supporting information.

## Conflict of interest

The authors declare that they have no known competing financial interests or personal relationships that could have appeared to influence the work reported in this paper.
