# Supplementary material for: Paxillin family proteins Hic-5 and LPXN promote lipid storage by regulating the ubiquitination degradation of CIDEC
Source: J Biol Chem. 2023 Dec 28;300(2):105610. doi: 10.1016/j.jbc.2023.105610 (PMC10850781; doi:10.1016/j.jbc.2023.105610)
Supplement: Supporting information [file mmc1.docx]

**Table S1. List of the HTRF-based screening data in this study**

| **gene** | **relative change%** |
| --- | --- |
| RFFL | 49.09456295 |
| PCGF2 | 36.18475388 |
| TRIM55 | 35.4828772 |
| CBLB | 34.82805831 |
| BRPF3 | 34.20619147 |
| AMFR | 33.09300424 |
| DCUN1D4 | 32.73879983 |
| FBXO18 | 32.51722835 |
| SCEL | 32.2602457 |
| CHFR | 32.2113936 |
| RNF139 | 31.81603836 |
| RNF207 | 31.57770807 |
| MEFV | 31.3675896 |
| MEX3C | 30.65146929 |
| RNF41 | 28.99691723 |
| FBXL11 | 28.86885095 |
| DPF1 | 28.31904416 |
| CXXC1 | 28.08971095 |
| MARCHF6 | 27.17657874 |
| FBXL10 | 26.82030239 |
| BIRC6 | 26.73237888 |
| ASB11 | 26.34808654 |
| MLLT10 | 26.24364141 |
| CAND1 | 24.73158344 |
| FBXO38 | 24.63696685 |
| PRICKLE3 | 24.48349686 |
| PHF8 | 24.28585836 |
| UNK | 23.58820929 |
| RNFT2 | 23.54852368 |
| RNF103 | 23.34804242 |
| CCL20 | 23.03579631 |
| MGRN1 | 22.59828447 |
| C10ORF46 | 22.42730071 |
| BIRC7 | 21.56079796 |
| RNF165 | 19.23685432 |
| SOCS1 | 19.18179229 |
| TRAIP | 17.89205582 |
| TRAF7 | 17.73386786 |
| CCNF | 17.5562677 |
| THNSL2 | 17.32792135 |
| TRIML1 | 17.32448049 |
| RNF112 | 16.9180325 |
| TRIM22 | 16.43046497 |
| FBXL8 | 16.4136115 |
| RC3H2 | 16.34583672 |
| CALCOCO2 | 16.20819105 |
| VPS41 | 15.30728676 |
| SOCS7 | 15.26944002 |
| FBXW7 | 15.26857258 |
| FBXL2 | 14.2435877 |
| BIRC3 | 14.21224362 |
| FBXL20 | 14.12219066 |
| ZNF330 | 13.59946069 |
| ASB5 | 13.59060755 |
| FBXO43 | 13.44236801 |
| TRIM40 | 13.4382541 |
| FBXL12 | 13.13837537 |
| SH3RF2 | 12.72631201 |
| RAB40A | 12.61314649 |
| FBXL3 | 12.02635751 |
| RAB40AL | 11.58939075 |
| DCUN1D3 | 11.45293485 |
| FBXW11 | 10.86225497 |
| PHF16 | 10.71578732 |
| PHF15 | 10.67596453 |
| RAB40C | 10.47005736 |
| WHSC1 | 10.14404493 |
| SOCS2 | 9.942783308 |
| OIT3 | 8.132355234 |
| FBXO15 | 7.819496619 |
| PHF20L1 | 7.54008491 |
| LGR6 | 7.103396468 |
| FBXW8 | 7.006812377 |
| MTF2 | 6.615552396 |
| BFAR | 6.03757886 |
| RNF7 | 5.352583269 |
| TNFAIP3 | 5.166712835 |
| AKTIP | 5.048749633 |
| TRIM5 | 5.017017478 |
| ZFAND6 | 4.864108827 |
| FBXO11 | 4.470000764 |
| RNF5 | 4.230749398 |
| TRAF4 | 4.144590057 |
| MID1 | 3.89606507 |
| AEBP1 | 3.568528254 |
| TNFAIP3 | 3.5629502 |
| BARD1 | 3.258302395 |
| CBLC | 3.099139481 |
| MEX3D | 2.898851262 |
| TRIM8 | 2.586126937 |
| PXMP3 | 2.502541619 |
| MAP3K1 | 2.3645435 |
| PHF17 | 1.339864169 |
| PHF13 | 1.180707869 |
| SYVN1 | 0.616879978 |
| FBXL6 | -0.157548359 |
| PRICKLE1 | -0.372292572 |
| INTS12 | -0.845076129 |
| LOC652759 | -1.014443593 |
| RNF113B | -2.54317593 |
| RNF38 | -3.10431048 |
| ZNF592 | -3.782837763 |
| PATZ1 | -3.921366122 |
| RNF220 | -4.198803452 |
| RUFY1 | -4.60650234 |
| PPIL2 | -4.720017985 |
| ANAPC11 | -5.041492869 |
| PHF23 | -5.382307174 |
| CDC34 | -5.558553977 |
| RCHY1 | -7.57851248 |
| RNF25 | -8.514284106 |
| FBXO8 | -8.972577089 |
| TRIM26 | -8.975399453 |
| MARCHF8 | -10.1078517 |
| SOCS4 | -10.59334342 |
| RNF12 | -10.82460763 |
| TRIM41 | -11.6842073 |
| TRIM10 | -12.5624811 |
| ZNRF3 | -12.7296177 |
| SOCS5 | -14.15569185 |
| FBXO45 | -14.5591878 |
| RNF43 | -14.82398177 |
| TRIM65 | -14.84695192 |
| PHF12 | -16.09635617 |
| TRIM29 | -16.89590983 |
| RNF122 | -17.04805457 |
| MLL4 | -17.2052456 |
| WDR24 | -17.25102603 |
| RNF183 | -17.5353594 |
| TRIM62 | -17.60805575 |
| TRIM15 | -18.49830688 |
| MDM4 | -18.62277689 |
| PHRF1 | -18.71511506 |
| ASB2 | -18.90564128 |
| RAB40B | -20.04931698 |
| RNF113A | -20.16518663 |
| CISH | -20.19141277 |
| DCUN1D5 | -20.41406408 |
| MLL2 | -20.64073797 |
| PRICKLE4 | -21.20737982 |
| TRAF5 | -22.16200536 |
| TRIM75 | -22.82013507 |
| PHF6 | -23.09966513 |
| CHD4 | -23.60966478 |
| MARCHF2 | -23.9076649 |
| TRIM59 | -24.33944107 |
| DCUN1D2 | -24.45610416 |
| PHF19 | -24.63352391 |
| RNF167 | -25.63766778 |
| BRAP | -25.82014983 |
| FBXO7 | -26.18553702 |
| HRC | -26.58063664 |
| FSD1L | -27.62841642 |
| FBXO32 | -27.84832441 |
| MIB2 | -28.22999095 |
| TRIM56 | -28.56069443 |
| DPF2 | -29.03433799 |
| SH3RF1 | -29.41303272 |
| TRIM25 | -29.77456921 |
| AOF1 | -29.85250569 |
| BAHD1 | -30.25914195 |
| JARID1B | -30.54287601 |
| FBXL17 | -30.72435384 |
| PEX10 | -30.8115876 |
| PDZRN3 | -31.34283997 |
| TRIM38 | -31.8017012 |
| BAZ2B | -32.00132823 |
| ZSWIM2 | -32.27038628 |
| RFWD3 | -32.32307611 |
| PHF10 | -32.38796366 |
| TRIM28 | -32.39041336 |
| ZMYND11 | -32.70204076 |
| JHDM1D | -32.94210846 |
| TRIM58 | -33.58124625 |
| MKRN2 | -33.63682435 |
| ZFAND5 | -33.98068866 |
| ASB9 | -34.0004034 |
| ASB3 | -34.14180917 |
| WHSC1L1 | -34.18663384 |
| RNF157 | -34.2148604 |
| ZNF547 | -34.30638128 |
| TRIM33 | -34.59003313 |
| TRAF6 | -34.62083621 |
| PML | -34.73409023 |
| PHF14 | -35.05455756 |
| HR | -35.1103275 |
| TRIM24 | -35.19057736 |
| PJA2 | -35.40261142 |
| TRIM54 | -36.54402919 |
| FBXL22 | -36.95463351 |
| RNF125 | -37.08562103 |
| MID2 | -37.79923583 |
| BRPF1 | -38.09223189 |
| TRAF3 | -38.21506838 |
| TRIM14 | -38.5593214 |
| CBL | -38.76002737 |
| MDM2 | -40.02185227 |
| RNF14 | -40.73233084 |
| PHF20 | -41.52107785 |
| MYCBP2 | -42.09464453 |
| VPS8 | -42.48609536 |
| RNF17 | -42.63206634 |
| JARID1D | -43.41602365 |
| CGRRF1 | -43.62673257 |
| RNF10 | -44.82108947 |
| PRPF19 | -45.15413845 |
| RNF216 | -45.29400978 |
| RSF1 | -45.52702514 |
| VPS11 | -45.6397431 |
| TRIM46 | -45.73906591 |
| RNF180 | -46.39523335 |
| MNAT1 | -46.41567438 |
| PDC | -47.27901772 |
| PHF1 | -47.66686451 |
| LPXN | -72.67945964 |

**
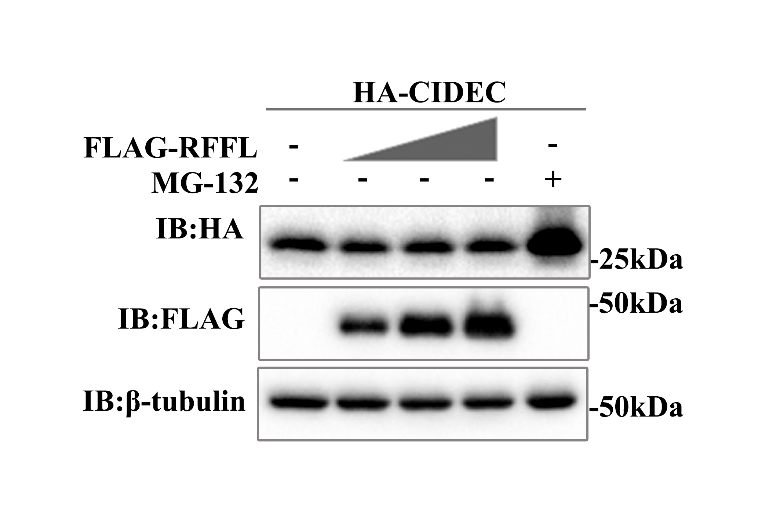
**

**Figure S1 Effect of RFFL on the protein level of CIDEC.**

HA-CIDEC was co-transfected with different amounts of FLAG-RFFL into 293T Cells.

The protein levels were analyzed by Western blotting.

**
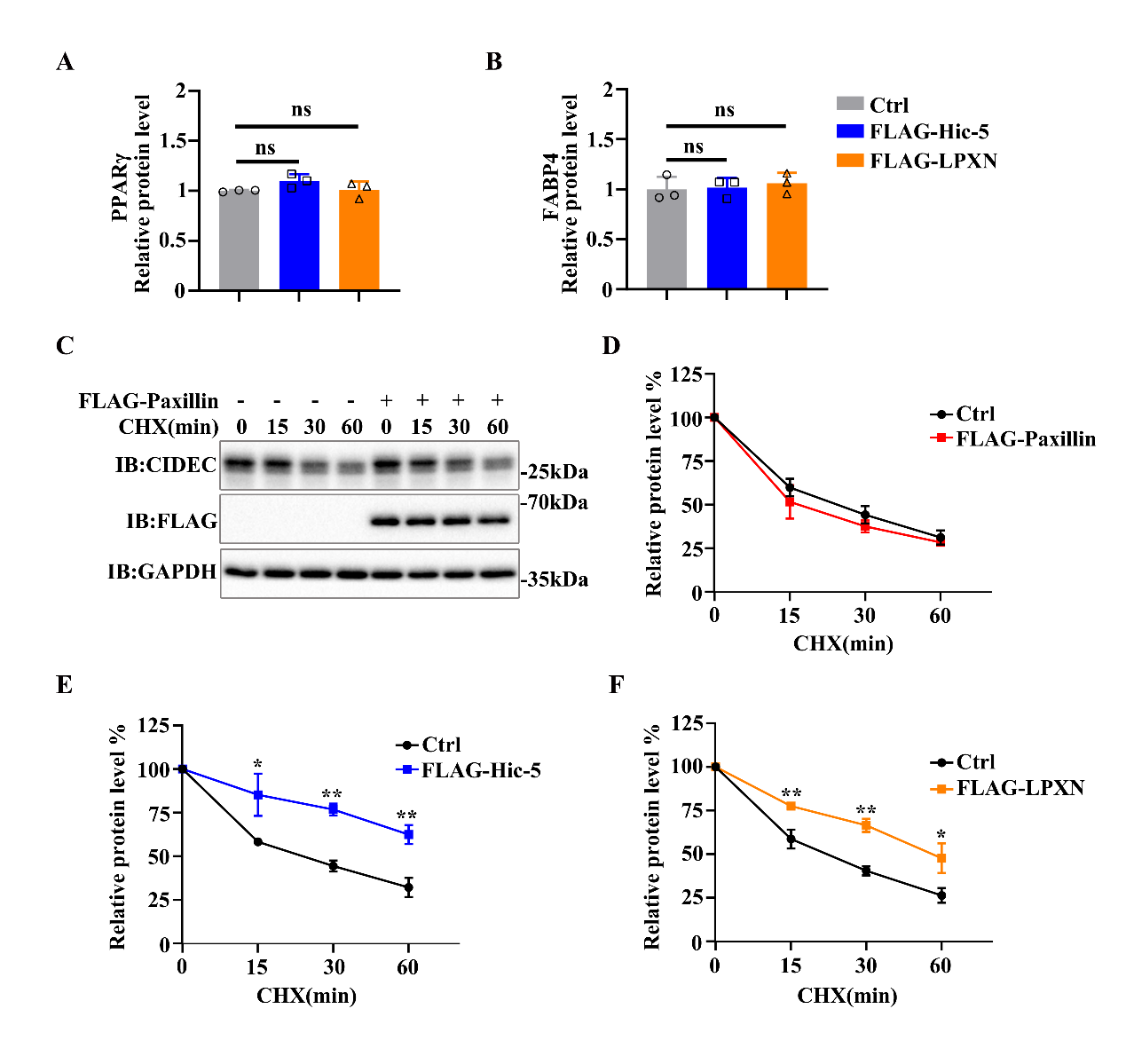
**

**Figure S2 Hic-5 and LPXN enhance the stability of CIDEC in adipocytes.**

(A-B) Quantitative analysis of relative protein level of PPARγ and FABP4 based on the results in (Figure 6A). (A Student’s two-tailed t test (unpaired)) for statistical analysis (*, p＜0.05; ***, p＜0.001; n=3). (C) Effect of Paxillin on the CIDEC stability. Differentiated 3T3-L1 cells (day 6 after induction) were infected with lentivirus encoding FLAG-tagged Paxillin for 2 days. Cells were harvested and the protein level was evaluated with Western blotting after the addition of CHX (100 μg/mL) for 0, 15, 30, or 60 min. (D) Quantitative analysis of the relative level of CIDEC from (C). (A Student’s two-tailed t test (unpaired)) for statistical analysis (*, p＜0.05; ***, p＜0.001; n=3). (E-F) Quantitative analysis of the relative level of CIDEC from (Figure 6C-D). (A Student’s two-tailed t test (unpaired)) for statistical analysis (*, p＜0.05; ***, p＜0.001; n=3).


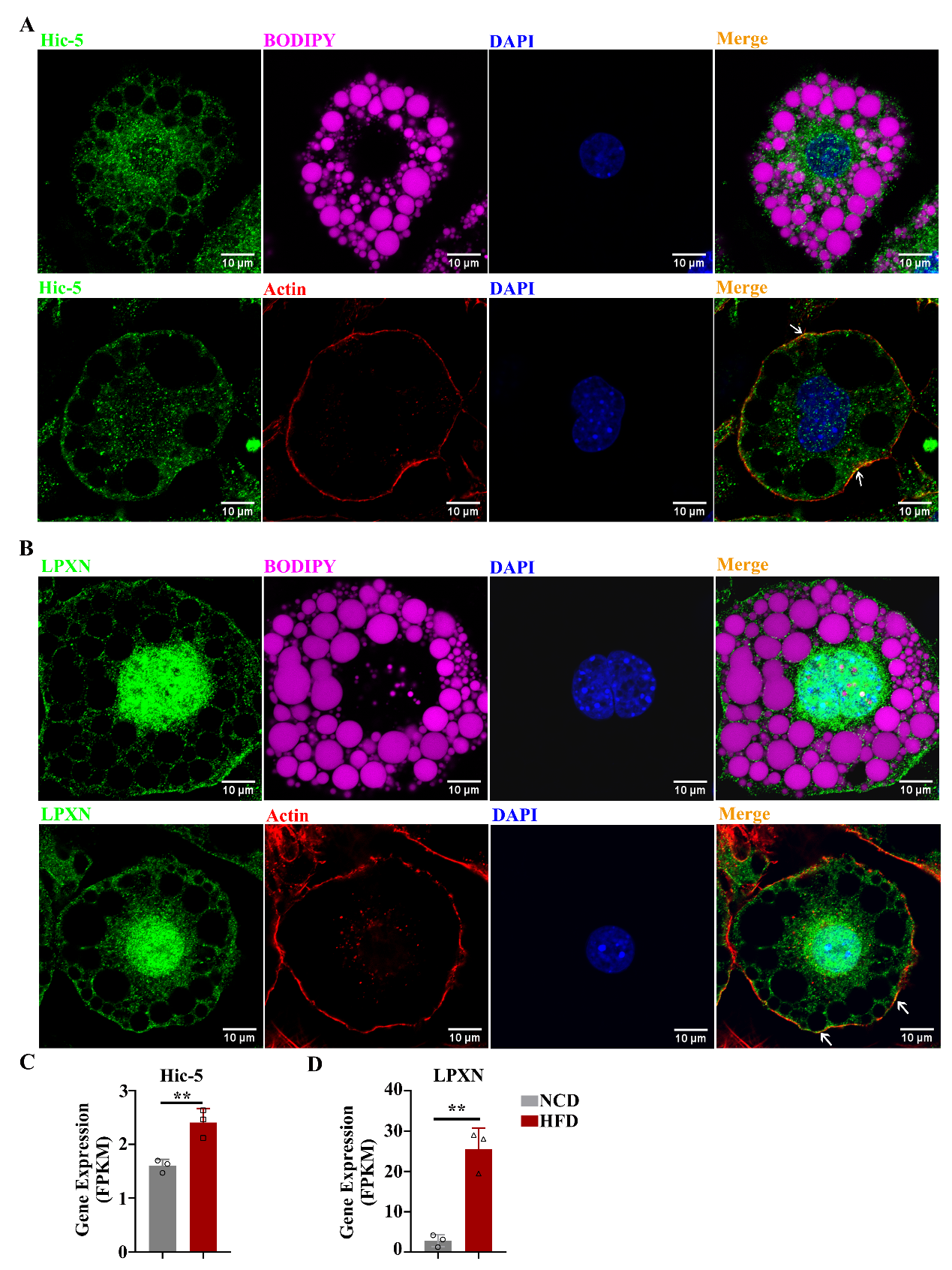


**Figure S3. The subcellular localization and expression levels** **of Hic-5 and LPXN.**

(A-B) The subcellular localization of Hic-5 and LPXN in adipocytes. Hic-5 was stained with anti-Hic-5 antibody (green), LPXN was stained with anti-LPXN antibody (green), LDs were stained with BODIPY 665 (magenta), actin was stained with phalloidin (red) and nuclei were stained with DAPI (blue). Scale bars, 10 μm. (C-D) Quantitative analysis of expression levels of Hic-5 and LPXN from the GEO database with accession number GSE182930. (A Student’s two-tailed t test (unpaired)) for statistical analysis (**, p＜0.01; n=3).
